# A “Non-Poisonous, Non-Irritating Dressing”

**Published:** 1890-12

**Authors:** 


### "A NON-POISONOUS NON-IRRITATING DRESSING."

Francis T. Henston and Charles T. Tichborne in a preliminary communication, before the recent meeting of the British Medical Association, reported in the *British Medical Journal* of Nov. 8th, give the results of their experiments with sulphite of zinc dressing—covering a broad field of capital and minor operations. The results obtained were better than under any other form of dressing. The list of operations in which this dressing was employed comprises amputations of breast, excisions of knee-joint, radical cure of inguinal hernia, psoas abscess, parotid tumor, arthrectomy, ovariotomy, nephrolithotomy, removal of false cartilages from the knee joint, etc.

Wounds dressed with the sulphite of zinc gauze pursue a perfectly aseptic course ; and in none of the cases was there the slightest irritation from the dressing; even where the discharges from the wounds were very abundant. "When sulphite of sodium is mixed with sulphate of zinc, the sulphite of zinc is very slowly formed, but is ultimately all deposited, owing to the insoluble nature of the new formed salt. This phenomenon adapts it naturally and with ease to the permanent saturation of any fabric, as gauze or lint, and without the intervention of an adhesive material such as starch."

The gauze is prepared as follows : "First boil the gauze in water to thoroughly wash and sterilize it; then upon this gauze is poured a boiling solution of zinc sulphate and sodium sulphite in equivalent proportions" (i. e., "six parts of zinc sulphate to five and a quarter parts of sodium sulphite in solution"); when thoroughly mixed and saturated the whole is allowed to stand twelve hours. The zinc sulphate is deposited in and around the fibers of the fabric in microscopic crystals, but soft and even unctious to the feel." To remove all sodium sulphate which may remain, the gauze is passed twice under rollers submerged in water.

The value of this dressing as an antiseptic consists in its slow oxidization, thus destroying germs. It has the additional advantage of the zinc base, " which is shown by the efficacy of the zinc salts in embalming the dead, and rendering the tissue an inert and unchanging mass."

We hope the new dressing will prove a decided acquisition to the surgical armamentarium, free from the irritating properties of plain bichloric and alem-broth dressing.

